# Synthesis and Anti-Inflammatory Activity of Some O-Propargylated-N-acetylpyrazole Derived from 1,3-Diarylpropenones

**DOI:** 10.1155/2016/3156593

**Published:** 2016-01-11

**Authors:** Ashwani Kumar Dhingra, Bhawna Chopra, Rameshwar Dass, Sanjeev K. Mittal

**Affiliations:** ^1^Guru Gobind Singh College of Pharmacy, Yamuna Nagar, Haryana 135001, India; ^2^Department of Pharmacy, IK Gujral Punjab Technical University, Jalandhar, Punjab 144601, India; ^3^Department of Industrial Chemistry, Guru Nanak Khalsa College, Yamuna Nagar, Haryana 135001, India; ^4^IEC School of Pharmacy, IEC University, Baddi, Himachal Pradesh 173205, India

## Abstract

In search of novel effective potent therapeutic agents delivered by oral route for inflammation treatment, some novel O-propargylated-N-acetylpyrazole analogs (**5a–j**) were prepared by treating N-acetylpyrazole (**4a–j**) derived from 1,3-diarylpropenones (**3a–j**) with propargyl bromide. Claisen-Schmidt condensation of a series of substituted aryl ketones** 1** and benzaldehydes** 2** in glacial acetic acid afforded 1,3-diarylpropenones which on further treatment with hydrazine hydrate in acetic acid under reflux conditions afforded 1-acetyl-3,5-diaryl-4,5-dihydro(1H)pyrazoles (**4a–j**). The products were characterized by using spectroscopic techniques such as IR and NMR. In addition, the* in vivo* anti-inflammatory activity of the synthesized compounds was determined using the carrageenan-induced paw oedema method in rats.

## 1. Introduction

Inflammation is a protective attempt by the organism to remove the injurious stimuli and to initiate the healing process. Also, it has been reported to be associated with the onset of various cancers [1]. It is a dynamic process and can be classified as either acute or chronic [2]. Acute inflammation is the exudation of plasma proteins and fluids and the emigration of leukocytes. Chronic inflammation is inflammation of prolonged duration in which tissue destruction, active inflammation, and attempts at repair are proceeding simultaneously [2]. Current approaches to overcome the inflammation include the use of nonsteroidal anti-inflammatory drugs (NSAIDS), immune selective anti-inflammatory derivatives, selective glucocorticoid receptor agonist, resolvins/protectins, and TNF inhibitors. Although drug treatment has been improved to some extent yet, it is still a challenge for the pharmaceutical chemists to explore the more effective and potent therapeutic agents to treat inflammation and reduce the signs and symptoms of acute inflammation and chronic inflammatory diseases. Thus, it is well evident from the literature and numerous studies that there is requirement for appropriate modification of the molecules to attenuate the effective potent therapeutic agents for the treatment of inflammation and also ensure that the host immune defense against infection is not impaired [1].

The chemistry of chalcones has generated an intensive scientific interest due to their wide spectrum of biological properties such as antibacterial [3], antifungal, insecticidal, anaesthetic, anti-inflammatory, analgesic, and ulcerogenic properties. Some substituted chalcones and their derivatives, including heterocyclic analogues, have been reported to possess some interesting biological properties [4–7] which are detrimental to the growth of microbes tubercle bacilli, malarial parasites, acrus,* Schistosoma*, and intestinal worms. Some of the compounds are also claimed to be toxic for animals and insect and are also reported to exhibit inhibitory action on several enzymes, fungi, and herbaceous plants.

1-Acetyl-3,5-diaryl-4,5-dihydro(1H)pyrazoles have previously been reported to exhibit a variety of biological activities such as inhibitors of monoamine oxidases, swine kidney oxidase and bovine serum amine oxidases [8, 9], anticancer via binding to P-glycoprotein [10, 11], anti-*Helicobacter pylori* [12], antiviral [13], antibacterial via inhibition of FabH [14], and anti-inflammatory [15, 16]. In the present paper, we describe the synthesis of some substituted N-acetylpyrazoles (**4a–j**) and their corresponding O-propargylate analogs (**5a–j**) as potent anti-inflammatory agents.

## 2. Materials and Methods

### 2.1. Chemistry

All melting points were determined by open capillary tube method and are uncorrected. IR spectra are recorded on Perkin Elmer RX1 spectrophotometer using KBr pellets and are expressed in cm^−1^. The ^1^HNMR spectra were recorded on Bruker 300 MHz spectrometer in (CDCl_3_) using TMS as an internal reference and chemical shifts were measured in *δ* ppm. The progress of the reaction was monitored by TLC using 0.2 mm thickness aluminium sheet precoated with silica gel Merck 60F 254 and visualization was done using iodine/UV lamp for detection of the spots. The solvent was removed under reduced pressure using Buchi rotary evaporator.

### 2.2. General Procedure for the Synthesis of 1,3-Diarylpropenones (**3a–j**)

Concentrated sulfuric acid (2.5 mL) was added slowly with stirring into a solution of various substituted benzaldehydes (1 mmol) and acetophenones (1 mmol) in glacial acetic acid. The stirring was continued for 48 h keeping the temperature of reaction mixture below 20–25°C. The completion of the reaction was monitored by TLC. After the completion, the reaction mixture was poured onto ice cold water (100 mL) and extracted thrice with ethylacetate. The organic layer was pooled and concentrated under reduced pressure to get crude 1,3-diarylpropenones which was recrystallized from ethanol and further used for the next step.

### 2.3. General Procedure for the Synthesis of N-Acetylpyrazoles (**4a–j**)

A mixture of various 1,3-diarylpropenones (1 mmol) in acetic acid and hydrazine monohydrate 80% (2 mmol) was refluxed for 4 hours. The mixture was then poured onto ice cold water (50 mL) to get crude pyrazole analogs (**4a–j**), which were purified by crystallization with ethanol to afford pure title compounds.

### 2.4. General Procedure for the Synthesis of O-Propargylated-N-acetylpyrazoles (**5a–j**)

**Scheme 1:**
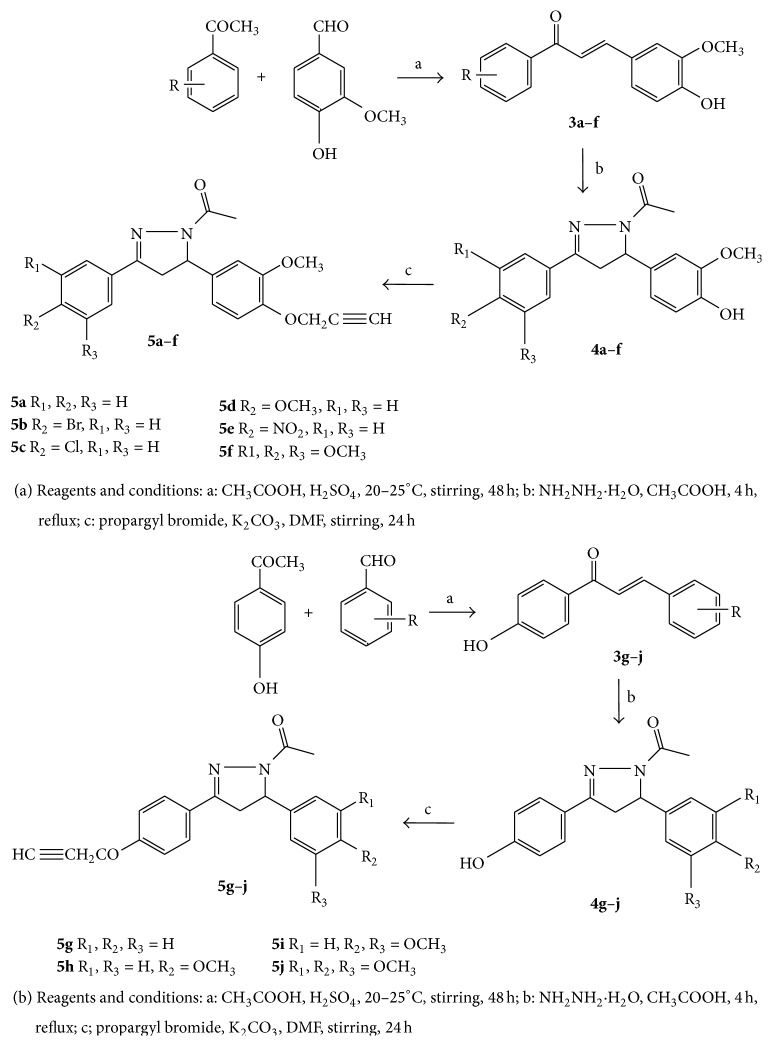
Synthetic methodology and structures of the all prepared analogs.

Propargyl bromide (1.5 mmol) was added to the stirred mixture of pyrazole (1 mmol) and K_2_CO_3_ (1.5 mmol) in dry DMF. The stirring was continued under anhydrous condition for 24 h keeping the temperature of reaction mixture below 5°C. After completion of reaction, as evident by TLC, the reaction mixture was poured onto ice cold water. Precipitates of O-propargylated-N-acetylpyrazole were then filtered out and dried. The remaining reactions were carried out following these general procedures. The structures of the all prepared analogs are depicted in Scheme 1.

### 2.5. Spectral Analysis of the Synthesized Compounds

#### 2.5.1. 1-(4,5-Dihydro-5-(4-hydroxy-3-methoxyphenyl)-3-phenylpyrazol-1-yl)ethanone** 4a**

Molecular Formula: C_18_H_18_N_2_O_3_; Yield: 80%; IR (KBr, cm^−1^): 1665 (C=O), 1630 (C=N), 1590 (aromatic C=C), 1265 (C–O), 1125 (C–N); ^1^HNMR (300 MHz, CDCl_3_, TMS = 0) *δ*: 1.8–2.0 (d, 2H, CH_2_), 2.1 (s, 3H, CH_3_), 3.7 (s, 3H, OCH_3_), 4.7 (t, 1H, CH), 5.1 (s, 1H, OH), 6.5–6.7 (m, 3H, Ar–H), 7.2–7.6 (m, 5H, Ar–H). ^13^C NMR (300 MHz, CDCl_3_): 168.3, 151.2, 143, 136.5, 134, 131.2, 128.4, 120.5, 115.7, 112.4, 59.3, 56.4, 39.2, 23.0.

#### 2.5.2. 1-(3-(4-Bromophenyl)-4,5-dihydro-5-(4-hydroxy-3-methoxyphenyl)pyrazol-1-yl)ethanone** 4b**

Molecular Formula: C_18_H_17_BrN_2_O_3_; Yield: 75%; IR (KBr, cm^−1^): 1660 (C=O), 1630 (C=N), 1590 (aromatic C=C), 1130 (C–N); ^1^HNMR (300 MHz, CDCl_3_, TMS = 0) *δ*: 1.9–2.1 (d, 2H, CH_2_), 2.1 (s, 3H, CH_3_), 3.6 (s, 3H, OCH_3_), 4.8 (t, 1H, CH), 5.0 (s, 1H, OH), 6.5–6.7 (m, 3H, Ar–H), 7.5–7.6 (m, 4H, Ar–H). ^13^C NMR (300 MHz, CDCl_3_): 168.5, 151.2, 143.4, 137.2, 133, 131.4, 125.4, 120.2, 116.3, 112.4, 59.3, 56.2, 39.4, 23.2.

#### 2.5.3. 1-(3-(4-Chlorophenyl)-4,5-dihydro-5-(4-hydroxy-3-methoxyphenyl)pyrazol-1-yl)ethanone** 4c**

Molecular Formula: C_18_H_17_ClN_2_O_3_; Yield: 70%; IR (KBr, cm^−1^): 1665 (C=O), 1630 (C=N), 1590 (aromatic C=C), 1265 (C–O), 1125 (C–N); ^1^HNMR (300 MHz, CDCl_3_, TMS = 0) *δ*: 1.8–2.0 (d, 2H, CH_2_), 2.0 (s, 3H, CH_3_), 3.7 (s, 3H, OCH_3_), 4.8 (t, 1H, CH), 5.0 (s, 1H, OH), 6.5–6.7 (m, 3H, Ar–H), 7.3–7.6 (m, 4H, Ar–H).

#### 2.5.4. 1-(4,5-Dihydro-5-(4-hydroxy-3-methoxyphenyl)-3-(4-methoxyphenyl)pyrazol-1-yl)ethanone** 4d**

Molecular Formula: C_19_H_20_N_2_O_4_; Yield: 75%; IR (KBr, cm^−1^): 1665 (C=O), 1630 (C=N), 1590 (aromatic C=C), 1265 (C–O), 1130 (C–N); ^1^HNMR (300 MHz, CDCl_3_, TMS = 0) *δ*: 1.8–2.0 (d, 2H, CH_2_), 2.1 (s, 3H, CH_3_), 3.6 (s, 6H, OCH_3_), 4.7 (t, 1H, CH), 5.0 (s, 1H, OH), 6.5–6.8 (m, 5H, Ar–H), 7.4–7.6 (m, 2H, Ar–H).

#### 2.5.5. 1-(4,5-Dihydro-5-(4-hydroxy-3-methoxyphenyl)-3-(4-nitrophenyl)pyrazol-1-yl)ethanone** 4e**

Molecular Formula: C_18_H_17_N_3_O_5_; Yield: 70%; IR (KBr, cm^−1^): 1660 (C=O), 1635 (C=N), 1590 (aromatic C=C), 1125 (C–N); ^1^HNMR (300 MHz, CDCl_3_, TMS = 0) *δ*: 1.9–2.1 (d, 2H, CH_2_), 2.2 (s, 3H, CH_3_), 3.7 (s, 3H, OCH_3_), 4.8 (t, 1H, CH), 5.1 (s, 1H, OH), 6.5–6.7 (m, 3H, Ar–H), 7.6–7.8 (m, 2H, Ar–H), 8.0–8.1 (m, 2H, Ar–H). ^13^C NMR (300 MHz, CDCl_3_): 168.2, 151.4, 143.2, 140.2, 137, 130.2, 121.4, 120.5, 116.5, 112.4, 59, 56.4, 39.1, 23.2.

#### 2.5.6. 1-(4,5-Dihydro-5-(4-hydroxy-3-methoxyphenyl)-3-(3,4,5-trimethoxyphenyl)pyrazol-1-yl)ethanone** 4f**

Molecular Formula: C_21_H_24_N_2_O_6_; Yield: 65%; IR (KBr, cm^−1^): 1665 (C=O), 1635 (C=N), 1590 (aromatic C=C), 1270 (C–O), 1130 (C–N); ^1^HNMR (300 MHz, CDCl_3_, TMS = 0) *δ*: 1.8–2.0 (d, 2H, CH_2_), 2.0 (s, 3H, CH_3_), 3.6 (s, 12H, OCH_3_), 4.7 (t, 1H, CH), 4.9 (s, 1H, OH), 6.5–6.6 (m, 5H, Ar–H).

#### 2.5.7. 1-(4,5-Dihydro-3-(4-hydroxyphenyl)-5-phenylpyrazol-1-yl)ethanone** 4g**

Molecular Formula: C_17_H_16_N_2_O_2_; Yield: 65%; IR (KBr, cm^−1^): 1660 (C=O), 1635 (C=N), 1595 (aromatic C=C), 1130 (C–N); ^1^HNMR (300 MHz, CDCl_3_, TMS = 0) *δ*: 1.8–2.0 (d, 2H, CH_2_), 2.1 (s, 3H, CH_3_), 4.8 (t, 1H, CH), 4.9 (s, 1H, OH), 6.7–7.3 (m, 9H, Ar–H). ^13^C NMR (300 MHz, CDCl_3_): 168, 160.4, 151.2, 143.0, 130.2, 128.2, 127, 126.4, 116.4, 58.1, 39.4, 23.4.

#### 2.5.8. 1-(4,5-Dihydro-3-(4-hydroxyphenyl)-5-(4-methoxyphenyl)pyrazol-1-yl)ethanone** 4h**

Molecular Formula: C_18_H_18_N_2_O_3_; Yield: 70%; IR (KBr, cm^−1^): 1665 (C=O), 1635 (C=N), 1590 (aromatic C=C), 1130 (C–N); ^1^HNMR (300 MHz, CDCl_3_, TMS = 0) *δ*: 1.8–2.0 (d, 2H, CH_2_), 2.0 (s, 3H, CH_3_), 3.7 (s, 3H, OCH_3_), 4.9 (t, 1H, CH), 5.0 (s, 1H, OH), 6.7–7.3 (m, 8H, Ar–H).

#### 2.5.9. 1-(4,5-Dihydro-3-(4-hydroxyphenyl)-5-(3,4-dimethoxyphenyl)pyrazol-1-yl)ethanone** 4i**

Molecular Formula: C_19_H_20_N_2_O_4_; Yield: 65%; IR (KBr, cm^−1^): 1660 (C=O), 1635 (C=N), 1595 (aromatic C=C), 1270 (C–O), 1130 (C–N); ^1^HNMR (300 MHz, CDCl_3_, TMS = 0) *δ*: 1.8–2.0 (d, 2H, CH_2_), 2.0 (s, 3H, CH_3_), 3.6 (s, 6H, OCH_3_), 4.9 (t, 1H, CH), 5.0 (s, 1H, OH), 6.6–7.4 (m, 7H, Ar–H).

#### 2.5.10. 1-(4,5-Dihydro-3-(4-hydroxyphenyl)-5-(3,4,5-trimethoxyphenyl)pyrazol-1-yl)ethanone** 4j**

Molecular Formula: C_20_H_22_N_2_O_5_; Yield: 65%; IR (KBr, cm^−1^): 1665 (C=O), 1635 (C=N), 1590 (aromatic C=C), 1270 (C–O), 1135 (C–N); ^1^HNMR (300 MHz, CDCl_3_, TMS = 0) *δ*: 1.9–2.1 (d, 2H, CH_2_), 2.1 (s, 3H, CH_3_), 3.7 (s, 9H, OCH_3_), 4.8 (t, 1H, CH), 5.0 (s, 1H, OH), 6.4–6.7 (m, 4H, Ar–H), 7.3–7.4 (m, 2H, Ar–H).

#### 2.5.11. 1-(4,5-Dihydro-5-(3-methoxy-4-(prop-2-ynyloxy)phenyl)-3-phenylpyrazol-1-yl)ethanone** 5a**

Molecular Formula: C_21_H_20_N_2_O_3_; Yield: 75%; IR (KBr, cm^−1^): 1665 (C=O), 1630 (C=N), 1590 (aromatic C=C), 1265 (C–O), 1125 (C–N); ^1^HNMR (300 MHz, CDCl_3_, TMS = 0) *δ*: 1.8–2.0 (d, 2H, CH_2_), 2.1 (s, 3H, CH_3_), 2.5 (s, 1H, CH), 3.7 (s, 3H, OCH_3_), 4.5 (s, 2H, CH_2_), 4.8 (t, 1H, CH), 6.7–7.5 (m, 8H, Ar–H).

#### 2.5.12. 1-(3-(4-Bromophenyl)-4,5-dihydro-5-(3-methoxy-4-(prop-2-ynyloxy)phenyl)pyrazol-1-yl)ethanone** 5b**

Molecular Formula: C_21_H_19_BrN_2_O_3_; Yield: 70%; IR (KBr, cm^−1^): 1660 (C=O), 1635 (C=N), 1595 (aromatic C=C), 1265 (C–O), 1130 (C–N); ^1^HNMR (300 MHz, CDCl_3_, TMS = 0) *δ*: 1.9–2.1 (d, 2H, CH_2_), 2.1 (s, 3H, CH_3_), 2.6 (s, 1H, CH), 3.6 (s, 3H, OCH_3_), 4.5 (s, 2H, CH_2_), 4.8 (t, 1H, CH), 6.6–7.5 (m, 7H, Ar–H).

#### 2.5.13. 1-(3-(4-Chlorophenyl)-4,5-dihydro-5-(3-methoxy-4-(prop-2-ynyloxy)phenyl)pyrazol-1-yl)ethanone** 5c**

Molecular Formula: C_21_H_19_ClN_2_O_3_; Yield: 60%; IR (KBr, cm^−1^): 1665 (C=O), 1635 (C=N), 1595 (aromatic C=C), 1265 (C–O), 1125 (C–N); ^1^HNMR (300 MHz, CDCl_3_, TMS = 0) *δ*: 1.8–2.0 (d, 2H, CH_2_), 2.0 (s, 3H, CH_3_), 2.5 (s, 1H, CH), 3.6 (s, 3H, OCH_3_), 4.6 (s, 2H, CH_2_), 4.8 (t, 1H, CH), 6.6–7.5 (m, 7H, Ar–H).

#### 2.5.14. 1-(4,5-Dihydro-5-(3-methoxy-4-(prop-2-ynyloxy)phenyl)-3-(4-methoxyphenyl)pyrazol-1-yl)ethanone** 5d**

Molecular Formula: C_22_H_22_N_2_O_4_; Yield: 70%; IR (KBr, cm^−1^): 1660 (C=O), 1635 (C=N), 1590 (aromatic C=C), 1265 (C–O), 1130 (C–N); ^1^HNMR (300 MHz, CDCl_3_, TMS = 0) *δ*: 1.8–2.0 (d, 2H, CH_2_), 2.0 (s, 3H, CH_3_), 2.6 (s, 1H, CH), 3.7 (s, 6H, OCH_3_), 4.6 (s, 2H, CH_2_), 4.8 (t, 1H, CH), 6.6–7.5 (m, 7H, Ar–H).

#### 2.5.15. 1-(4,5-Dihydro-5-(3-methoxy-4-(prop-2-ynyloxy)phenyl)-3-(4-nitrophenyl)pyrazol-1-yl)ethanone** 5e**

Molecular Formula: C_21_H_19_N_3_O_5_; Yield: 65%; IR (KBr, cm^−1^): 1660 (C=O), 1635 (C=N), 1590 (aromatic C=C), 1125 (C–N); ^1^HNMR (300 MHz, CDCl_3_, TMS = 0) *δ*: 1.8–2.0 (d, 2H, CH_2_), 2.1 (s, 3H, CH_3_), 2.5 (s, 1H, CH), 3.7 (s, 3H, OCH_3_), 4.6 (s, 2H, CH_2_), 4.9 (t, 1H, CH), 6.6–6.7 (m, 3H, Ar–H), 7.7–7.8 (m, 2H, Ar–H), 8.1–8.2 (m, 2H, Ar–H). ^13^C NMR (300 MHz, CDCl_3_): 168.2, 151.4, 150.5, 149.6, 147.2, 140.1, 136.2, 130.2, 121.3, 120.2, 115.4, 112, 78.1, 59, 57.2, 39.1, 23.1.

#### 2.5.16. 1-(4,5-Dihydro-5-(3-methoxy-4-(prop-2-ynyloxy)phenyl)-3-(3,4,5-trimethoxyphenyl)pyrazol-1-yl)ethanone** 5f**

Molecular Formula: C_24_H_26_N_2_O_6_; Yield: 60%; IR (KBr, cm^−1^): 1665 (C=O), 1635 (C=N), 1590 (aromatic C=C), 1270 (C–O), 1130 (C–N); ^1^HNMR (300 MHz, CDCl_3_, TMS = 0) *δ*: 1.8–2.0 (d, 2H, CH_2_), 2.0 (s, 3H, CH_3_), 2.5 (s, 1H, CH), 3.7 (s, 12H, OCH_3_), 4.6 (s, 2H, CH_2_), 4.9 (t, 1H, CH), 6.6–6.7 (m, 5H, Ar–H).

#### 2.5.17. 1-(4,5-Dihydro-5-phenyl-3-(4-(prop-2-ynyloxy)phenyl)pyrazol-1-yl)ethanone** 5g**

Molecular Formula: C_20_H_18_N_2_O_2_; Yield: 65%; IR (KBr, cm^−1^): 1660 (C=O), 1635 (C=N), 1595 (aromatic C=C), 1130 (C–N); ^1^HNMR (300 MHz, CDCl_3_, TMS = 0) *δ*: 1.8–2.0 (d, 2H, CH_2_), 2.0 (s, 3H, CH_3_), 2.5 (s, 1H, CH), 4.5 (s, 2H, CH_2_), 4.9 (t, 1H, CH), 6.8–7.4 (m, 9H, Ar–H).

#### 2.5.18. 1-(4,5-Dihydro-5-(4-methoxyphenyl)-3-(4-(prop-2-ynyloxy)phenyl)pyrazol-1-yl)ethanone** 5h**

Molecular Formula: C_21_H_20_N_2_O_3_; Yield: 70%; IR (KBr, cm^−1^): 1665 (C=O), 1635 (C=N), 1590 (aromatic C=C), 1130 (C–N); ^1^HNMR (300 MHz, CDCl_3_, TMS = 0) *δ*: 1.8–2.0 (d, 2H, CH_2_), 2.1 (s, 3H, CH_3_), 2.4 (s, 1H, CH), 3.7 (s, 3H, OCH_3_), 4.5 (s, 2H, CH_2_), 4.8 (t, 1H, CH), 6.7–7.5 (m, 8H, Ar–H).

#### 2.5.19. 1-(4,5-Dihydro-5-(3,4-dimethoxyphenyl)-3-(4-(prop-2-ynyloxy)phenyl)pyrazol-1-yl)ethanone** 5i**

Molecular Formula: C_22_H_22_N_2_O_4_; Yield: 65%; IR (KBr, cm^−1^): 1660 (C=O), 1635 (C=N), 1595 (aromatic C=C), 1270 (C–O), 1130 (C–N); ^1^HNMR (300 MHz, CDCl_3_, TMS = 0) *δ*: 1.8–2.0 (d, 2H, CH_2_), 2.0 (s, 3H, CH_3_), 2.5 (s, 1H, CH), 3.7 (s, 6H, OCH_3_), 4.6 (s, 2H, CH_2_), 4.9 (t, 1H, CH), 6.6–6.8 (m, 5H, Ar–H), 7.4–7.5 (m, 2H, Ar–H).

#### 2.5.20. 1-(4,5-Dihydro-5-(3,4,5-trimethoxyphenyl)-3-(4-(prop-2-ynyloxy)phenyl)pyrazol-1-yl)ethanone** 5j**

Molecular Formula: C_23_H_24_N_2_O_5_; Yield: 60%; IR (KBr, cm^−1^): 1660 (C=O), 1635 (C=N), 1595 (aromatic C=C), 1270 (C–O), 1135 (C–N); ^1^HNMR (300 MHz, CDCl_3_, TMS = 0) *δ*: 1.8–2.0 (d, 2H, CH_2_), 2.0 (s, 3H, CH_3_), 2.6 (s, 1H, CH), 3.7 (s, 9H, OCH_3_), 4.6 (s, 2H, CH_2_), 4.9 (t, 1H, CH), 6.4–6.8 (m, 4H, Ar–H), 7.3–7.4 (m, 2H, Ar–H).

### 2.6. Biological Activity

#### 2.6.1. Animals and Instruments

Adult Wistar rats of either sex weighing within 150–180 g were used throughout the work. The selected animals were kept under standard conditions of light and temperature with free access to food and water. All experimental procedures were carried out in strict accordance with the guidelines prescribed by the committee for the purpose of control and supervisions on experimentation on animals (CPCSEA) and were approved by the Institutional Animal Ethics Committee of Guru Gobind Singh College of Pharmacy, Yamuna Nagar, Haryana (Regn. Number 873/PO/ac/05/CPCSEA). The paw edema was induced by subplantar injection using carrageenan and the increased foot volumes were measured in a plethysmograph by water displacement.

#### 2.6.2. Anti-Inflammatory Activity

The anti-inflammatory activity was carried out by carrageenan-induced paw edema test [17]. The animals were randomly divided into twenty-two groups of six rats each. Test compounds (**4a–i** and** 5a–i**) and standard drug indomethacin were suspended in 0.5% w/v of sodium carboxyl methylcellulose (CMC), which was used as a vehicle for the control group. The rats were dosed with test drugs orally (100 mg/kg body weight) including the reference standard (10 mg/kg body weight) with help of oral catheter. After 30 minutes of drug administration, 0.1 mL of 1% w/v carrageenan solution in saline (0.9%) was injected in the subplantar region of the left hind paw of control as well as standard and test groups. The increased volume of paw edema (in mL) was determined immediately after injection of carrageenan and 4 h later. The difference between these two values was taken as edema volume. The percentage protection against inflammation was calculated as follows: (1 − *Vd*)/*Vc* × 100, where *Vc* is the increase in paw volume in the absence of the test compound (control) and *Vd* is the increase of paw volume after injection of the test compound. Data were expressed as means ± SEM. Significant differences between the control and treated groups were obtained using Dunnett's test with *p* value <0.05.

## 3. Result and Discussion

### 3.1. Chemistry

Claisen-Schmidt condensation of substituted aryl ketones (**1**) and benzaldehydes (**2**) in glacial acetic acid and H_2_SO_4_ yielded 1,3-diarylpropenones (Scheme 1,** 3a–j**; 70–90%) which on further treatment with hydrazine hydrate in presence of acetic acid under reflux afforded N-acetylpyrazolines (Scheme 1;** 4a–j**; 65–80%). These pyrazoles analogs were further treated with propargyl bromide and K_2_CO_3_ in dry DMF affords title O-propargylated-N-acetylpyrazole (Scheme 1,** 5a–j**, 60–75%) after recrystallization in ethanol (Schemes 1(a) and 1(b)). The purity and structures of all the synthesized compounds have been elucidated on the basis of their spectral data including IR and ^1^HNMR.

### 3.2. Anti-Inflammatory Activity

The* in vivo* anti-inflammatory activity was studied using carrageenan-induced rat paw edema model. The anti-inflammatory activity of all the prepared analogs along with standard drug indomethacin is depicted in Table 1. The test and standard drug produced significant inhibition of paw edema as compared to control. Out of all prepared analogs,** 4e**,** 4g**,** 5e**,** 4b,** and** 4a** exhibit a comparable activity to that of standard drug indomethacin.

## 4. Conclusions

In summary, the present investigation describes synthesis and anti-inflammatory potential of some O-propargylated-N-acetylpyrazole (**5a–j**) along with N-acetylpyrazole analogs (**4a–j**) derived from 1,3-diarylpropenones (**3a–j**), which was prepared by condensation of various substituted benzaldehydes and acetophenones. The prepared compounds were characterized by suitable methods such as spectroscopic evaluation like IR, ^1^HNMR, and ^13^C NMR. All spectral data were in accordance with assumed structures. The prepared analogs show remarkable reduction in inflammation, after 4 h of carrageenan administration as compared to control. Out of all prepared analogs, five compounds (**4e**,** 4g**,** 5e**,** 4b,** and** 4a)** delivered by oral route exhibit a comparable anti-inflammatory activity to that shown with indomethacin, suggesting a good oral bioavailability and a potent effect on inflammation. The promising activity of these compounds related to their structures can also be useful for establishing the structure activity relationship studies. SAR study revealed that nature of substituent(s) particularly electron withdrawing groups on aromatic ring greatly affects the anti-inflammatory activity. These novel compounds will be further examined upon an oral repeated oral delivery for their toxicity to verify their potential therapeutic effect.

## Figures and Tables

**Table 1:** Anti-inflammatory potential of test compounds (**4a–j** and **5a–j**) on carrageenan-induced rat paw edema (mL), % protection, and relative activity to indomethacin.

Tested compounds	Increase in paw edema (mL) ± SEM^a,b^	% protection	Relative activity to indomethacin
Control	0.94 ± 0.018	0.0	0.0
Indomethacin	0.24 ± 0.015	74.5	100
**4a**	0.23 ± 0.023	75.5	101.3
**4b**	0.23 ± 0.014	75.5	101.3
**4c**	0.27 ± 0.021	71.3	95.7
**4d**	0.64 ± 0.019	31.9	42.8
**4e**	0.21 ± 0.017	77.7	104.2
**4f**	0.76 ± 0.024	19.1	25.6
**4g**	0.22 ± 0.012	76.6	102.8
**4h**	0.55 ± 0.021	41.5	55.7
**4i**	0.54 ± 0.016	42.6	57.2
**4j**	0.68 ± 0.029	24.4	32.8
**5a**	0.38 ± 0.020	59.6	80
**5b**	0.32 ± 0.027	66.0	88.6
**5c**	0.37 ± 0.021	60.6	81.3
**5d**	0.71 ± 0.015	24.5	32.9
**5e**	0.22 ± 0.013	76.6	102.8
**5f**	0.78 ± 0.014	17.0	22.8
**5g**	0.43 ± 0.022	54.3	72.9
**5h**	0.73 ± 0.022	22.3	29.9
**5i**	0.71 ± 0.023	24.4	32.8
**5j**	0.75 ± 0.022	20.2	27.1

^a^SEM denotes the standard error of the mean. ^b^All data are significantly different from control (*p* < 0.05).
